# Early- to Late-Life Environmental Factors and Late-Life Global Cognition: The SONIC study

**DOI:** 10.1093/geroni/igaa057.1169

**Published:** 2020-12-16

**Authors:** Yoshiko Ishioka, Yasuyuki Gondo, Yukie Masui, Takeshi Nakagawa, Madoka Ogawa, Hiroki Inagaki, Saori Yasumoto, Tatsuro Ishizaki

**Affiliations:** 1 Keio University, Minato-ku, Kanagawa, Japan; 2 Osaka University, Suita, Osaka, Japan; 3 Tokyo Metropolitan Institute of Gerontology, Itabashi-ku, Tokyo, Japan; 4 National Center for Geriatrics and Gerontology, Ōbu, Aichi, Japan

## Abstract

Life environment across the life course—such as engagement in late-life leisure activity (LA), primary occupation, and early-life education—have been reported to be associated with better late-life cognitive outcomes. However, few studies have included all these factors from the past to the present due to the time-consuming procedure to measure all factors. This study examined (1) whether late-life LA is associated with better late-life cognition, after considering other life environments and (2) whether occupation, education, and childhood intelligence quotient have indirect effects on the late-life cognition through late-life LA. We used baseline data from the groups of 70- and 80-year-olds in the SONIC study (N = 1721 ). Global cognition was measured using the Montreal Cognitive Assessment. As for LA, participants were asked for yes/no answers to questions regarding their engagement in 158 activities. A latent factor representing LA was used in the analyses. We retrospectively evaluated the complexity of work with data, people, and things. As early-life environments, education and language and arithmetic abilities during elementary school were included in the analyses. Age and gender were controlled. A structural equation model showed that late-life LA was significantly associated with higher global cognition, even after controlling for all past factors (RMSEA = .050, GFI = .973, AGFI = .947). Sobel tests showed significant indirect effects of occupation, education, and childhood abilities on cognitive function. Results were robust across age and gender. It is suggested that engagement in LA explains individual differences in late-life cognitive function.

